# Effects of Preferred Music on Pain Tolerance During an Experimental Cold Pressor Test: A Pilot Study

**DOI:** 10.7759/cureus.101397

**Published:** 2026-01-12

**Authors:** Henry Francisco, Udoka Okpalauwaekwe, Stephan Milosavljevic

**Affiliations:** 1 Rehabilitation Medicine, University of Saskatchewan, Saskatoon, CAN; 2 Academic Family Medicine, University of Saskatchewan, Saskatoon, CAN

**Keywords:** cold pressor test, experimental pain, music-induced analgesia, non-pharmacologic pain management, pain perception, pain tolerance

## Abstract

Background

Preferred music has shown promise as a non-pharmacologic strategy for modulating pain, yet its effects in controlled laboratory environments relevant to rehabilitation practice remain underexplored. This pilot study examined whether listening to self-selected preferred music influences pain perception during an experimental cold pressor task.

Methods

Twenty healthy adults (mean age: 35.4 years) completed six cold pressor cycles in a repeated-measures design: two pre-music trials, two preferred-music trials, and two post-music trials. During each trial, participants immersed their non-dominant hand in 0°C water until the first pain perception. Outcomes included time-to-pain perception (seconds) and pain intensity (10-cm Visual Analogue Scale). Friedman’s test with Wilcoxon post-hoc comparisons analyzed non-normally distributed time-to-pain data, while repeated-measures ANOVA evaluated normally distributed pain intensity scores.

Results

Preferred music significantly increased pain tolerance. Mean time-to-pain perception rose from 33.8 s (pre-music) to 56.2 s during preferred-music trials, decreasing to 45.4 s post-music. The overall effect of the condition was significant (χ²(2) = 15.6, p < .001). Post-hoc tests showed significantly greater tolerance during preferred-music trials compared with both pre-music (p = 0.001) and post-music (p = 0.001) conditions, with 17 of 20 participants demonstrating increased tolerance. Pain intensity ratings did not differ significantly across conditions (F(2, 38) = 0.561, p > 0.05).

Conclusion

Preferred music meaningfully delayed pain onset during the cold pressor test (CPT) but did not alter perceived pain intensity. These findings support preferred music as a simple, low-cost strategy that may enhance pain tolerance and patient engagement during rehabilitation activities. Larger, clinically focused studies are needed to clarify mechanisms, the durability of effects, and real-world therapeutic applications.

## Introduction

Chronic pain is a major global health challenge, affecting more than 30% of the world’s population and contributing significantly to disability, reduced quality of life, and increased healthcare utilization [1]. Although opioid pharmacotherapy remains a common treatment approach, its use is associated with substantial risks, including misuse, dependence, and overdose [2,3]. As such, these risks have intensified a search for safe, accessible, and non-pharmacologic strategies to support pain management.

An emerging adjunctive approach is music-induced analgesia (MIA), defined as the pain-relieving effect of listening to music [4-6]. Evidence suggests that music may modulate pain perception through cognitive, emotional, and neurophysiological mechanisms [4-8]. Experimental studies have shown that preferred music (i.e., music chosen or preferred by the listener) may increase pain thresholds and decrease perceived pain during controlled nociceptive tasks [6,7,9]. For instance, Timmerman et al. and Van der Valk Bouman et al. demonstrated that preferred music significantly increased pain tolerance during cold pressor paradigms involving hundreds of healthy volunteers [9,10]. Similarly, Garcia and Hand found that music preference contributes to emotional regulation and distraction, enhancing analgesic effects [4].

Despite growing evidence, several gaps remain. Much of the existing literature has involved large festival-based samples or heterogeneous experimental pain stimuli, and relatively few studies have investigated preferred music analgesia in controlled, laboratory-like environments linked to rehabilitation settings. Moreover, although systematic reviews [11] support the potential of music as an adjunct to pain management, the specific mechanisms (such as modulation within affective, cognitive, and reward pathways) are still being clarified [12,13]. As rehabilitation professionals increasingly seek practical, low-cost strategies to support patient engagement and comfort, understanding the analgesic potential of preferred music during clinically relevant stimuli (e.g., cold pressor tasks) remains important.

To address this gap, our present pilot study examined whether listening to self-selected preferred music alters time-to-pain perception and perceived pain intensity during an experimental cold pressor test (CPT). We hypothesized that, compared with no-music conditions, preferred music would significantly increase the time to reach pain perception and reduce reported pain intensity.

Study objectives

This pilot study aims to evaluate the analgesic effects of preferred music during an experimental CPT among healthy adults. Specifically, we aimed to (a) compare time-to-pain perception between no-music and preferred-music conditions; (b) compare pain intensity self-ratings between no-music and preferred-music conditions; and (c) determine whether preferred music significantly increases pain tolerance and/or reduces perceived pain intensity.

## Materials and methods

Study design

This pilot study used a repeated-measures experimental design to examine the effect of preferred music on pain perception during a CPT. Data collection occurred between May 15, 2024, and May 15, 2025, in a controlled laboratory environment. A CPT involves the immersion of a limb (typically the hand) in near-freezing water, inducing both acute pain and physiological stress responses [14]. CPTs are widely used to assess pain perception and tolerance due to their reliability, safety, and sensitivity to physiological and psychological modulators of pain [14-16]. For our pilot study, each participant completed six cold pressor cycles under two experimental conditions: no-music and preferred-music exposure. Outcomes were measured within-person across conditions.

Participants and recruitment

Participants were recruited through self-identification in response to digital advertisements posted on the University of Saskatchewan websites. Interested individuals received an information sheet and were screened for eligibility prior to enrolment.

Eligibility criteria

Participants were eligible for inclusion if they were healthy adults between 18 and 65 years and were able to provide informed consent. Individuals were excluded if they reported any acute or chronic pain at the time of participation, were taking medications that could influence cold sensation, or had a diagnosed neurological disorder, cardiovascular disease, hemophilia, cancer, or any communicable or infectious condition. All potential participants younger than 18 or older than 65 were also excluded to ensure safety and consistency with established parameters for the cold pressor protocols [14,16].

Ethical considerations

Ethics approval was obtained from the University of Saskatchewan Behavioural Research Ethics Board (No. 4761). All participants consented prior to the commencement of this study.

Experimental procedure

All testing took place in a controlled laboratory environment. Participants were seated comfortably beside two water containers positioned near their non-dominant hand: a warm water bath maintained at approximately 37°C (±1°C) and a cold pressor bath prepared with an ice-water mixture at approximately 0°C. To begin each cycle, participants immersed their non-dominant hand in the warm bath for five minutes. After this acclimatization period, they were instructed to immediately transfer the hand into the cold pressor bath and keep it submerged until the first moment at which pain was perceived. At the point of first perceived pain, participants removed their hand and returned it to the warm bath for a five-minute recovery period. Each participant completed a total of six cycles in this order: two pre-music cycles without music, followed by two cycles in which preferred music was delivered through noise-cancelling headphones, and ending with two post-music cycles without music. To minimize response bias, participants were not shown previous trial scores, and pain ratings were recorded on separate sheets after each cycle.

Outcome measures

Two primary outcomes were evaluated. Time-to-pain perception was measured in seconds using a handheld stopwatch, beginning at the moment the hand entered the cold pressor bath and ending when the participant verbally indicated that pain was first perceived. Pain intensity was assessed immediately after hand withdrawal using a 10-cm Visual Analogue Scale (VAS), anchored at 0 cm indicating “no pain” and 10 cm indicating “worst imaginable pain” [17]. Demographic characteristics (age, sex, and dominant hand) were also collected to contextualize participant variability.

Statistical analysis

Data analyses were conducted using Microsoft Excel (Microsoft Corp., Redmond, WA, USA) and IBM SPSS Statistics for Windows, Version 28.0 (Released 2021; IBM Corp., Armonk, NY, USA). Normality of outcomes was assessed prior to inferential testing. Time-to-pain perception was non-normally distributed; therefore, a Friedman’s test (non-parametric repeated-measures ANOVA) with Bonferroni correction (p < 0.017) was used. Post-hoc comparisons employed the Wilcoxon signed-rank test. Pain intensity was normally distributed; a one-way repeated-measures ANOVA with Bonferroni correction (p < 0.017) compared pre-music, music, and post-music conditions. Post-hoc comparisons used paired t-tests. Descriptive statistics (means, medians, standard deviations) summarized all variables. Our primary contrasts of interest were (a) preferred music vs pre-music (baseline) and (b) preferred music vs post-music.

## Results

Participant characteristics

Twenty healthy adults participated in the study (13 female (65%), seven male (35%); mean age 35.4 years, range 22-65). Demographics and individual values for all cold pressor trials are presented in Table 1. Most participants (18/20; 90%) were left-hand dominant, and one participant (5%) demonstrated exceptionally high tolerance values (>300 s), noted as an outlier in subsequent distribution assessments.

**Table 1:** Demographic characteristics and individual cold pressor responses for each condition

Age (years)	Gender	Hand	Time to pain (s)			Pain score (cms)		
			Pre-music	Music	Post-music	Pre-music	Music	Post-music
65	M	Right	9.4	10.9	11.5	3.6	4.5	4.3
29	M	Left	38.1	47.1	40.7	3.8	2.7	4.6
25	M	Left	11.1	11.2	10.9	4.9	4.5	2.4
55	F	Left	40.0	323.8	262.7	1.0	0.6	0.6
42	F	Left	26.8	33.3	27.7	2.7	2.1	1.8
34	M	Left	25.7	17.7	16.4	4.2	5.2	4.9
37	M	Left	58.6	84.9	40.5	3.3	3.3	4.4
25	F	Left	43.4	66.3	54.5	3.9	3.8	3.7
23	F	Left	40.0	44.6	42.0	2.1	1.4	1.4
53	F	Left	20.7	33.6	24.4	1.5	1.6	1.3
24	F	Left	51.0	66.0	53.6	5.8	4.2	6.4
40	F	Left	15.2	21.9	17.1	4.9	4.2	4.6
40	F	Left	43.5	40.3	41.1	6.1	6.6	7.2
22	F	Left	73.6	106.0	80.5	1.8	2.8	2.7
27	F	Left	49.9	54.9	36.5	1.6	1.9	4.0
27	M	Left	12.1	11.2	11.0	3.9	3.7	4.3
43	F	Left	12.1	17.0	16.6	6.7	6.1	6.6
31	F	Left	30.8	43.4	42.1	7.0	4.4	6.9
25	F	Right	61.8	73.5	68.9	1.6	1.6	0.9
40	M	Left	11.5	17.1	8.6	4.0	3.1	4.8

Time-to-pain perception

**Figure 1:**
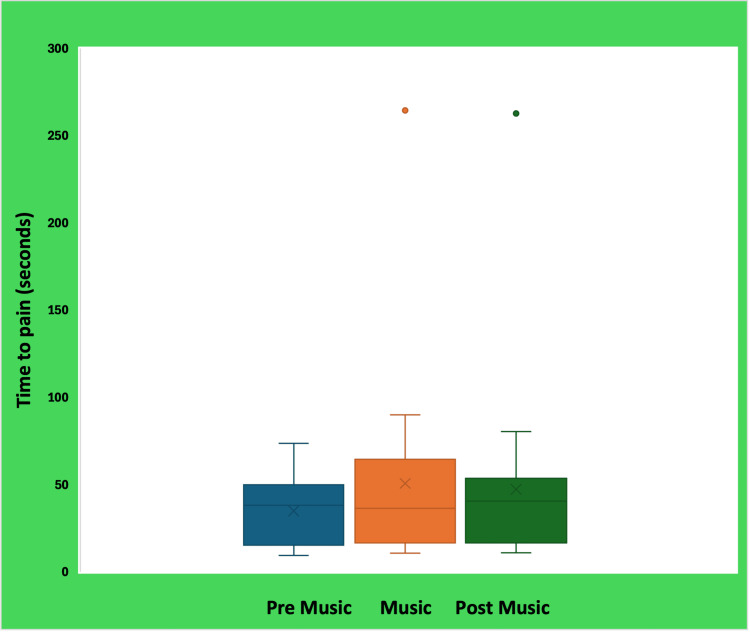
Time-to-pain perception during pre-music, preferred music, and post-music conditions The boxplots illustrate the distribution of time-to-first pain perception (s) across experimental conditions. The horizontal line within each box represents the median, the box indicates the interquartile range, and the whiskers denote the range of non-outlier values. The “×” symbol represents the mean value for each condition. Green and orange dots indicate individual outlier observations exceeding 1.5 times the interquartile range, reflecting participants with unusually high pain tolerance values during the music and post-music conditions.

Mean time-to-pain perception differed across the three conditions. As shown in Table 2, participants reached pain perception most quickly during the pre-music trials (mean = 33.8 s), exhibited substantially longer tolerance when listening to preferred music (mean = 56.2 s), and showed an intermediate response in the post-music trials (mean = 45.4 s). The distribution of scores for each condition is visually represented in Figure 1, which highlights the presence of outliers, particularly within the music and post-music conditions.

**Table 2:** Mean time-to-pain and mean pain scores for each condition

Variable	Pre-music	Music	Post-music
Time to pain (s), mean (SD)	33.8 (19.2)	56.2 (68.4)	45.4 (55.0)
Pain score (cm), mean (SD)	3.7 (1.8)	3.4 (1.6)	3.9 (2.0)

Non-parametric analysis using the Friedman test revealed a significant difference in time-to-pain perception across the three conditions (χ²(2) = 15.6, p < .001). Post-hoc Wilcoxon signed-rank tests with a Bonferroni-adjusted alpha of 0.017 confirmed that preferred music significantly increased pain tolerance when compared with both the pre-music (p = 0.001) and post-music (p = 0.001) conditions. No difference was observed between the two no-music conditions (pre-music vs. post-music; p = 0.232). These findings indicate that preferred music produced a robust and immediate effect, enhancing pain tolerance for the majority of participants; 17 out of 20 individuals (85%) demonstrated longer time-to-pain perception during the music condition.

**Figure 2:**
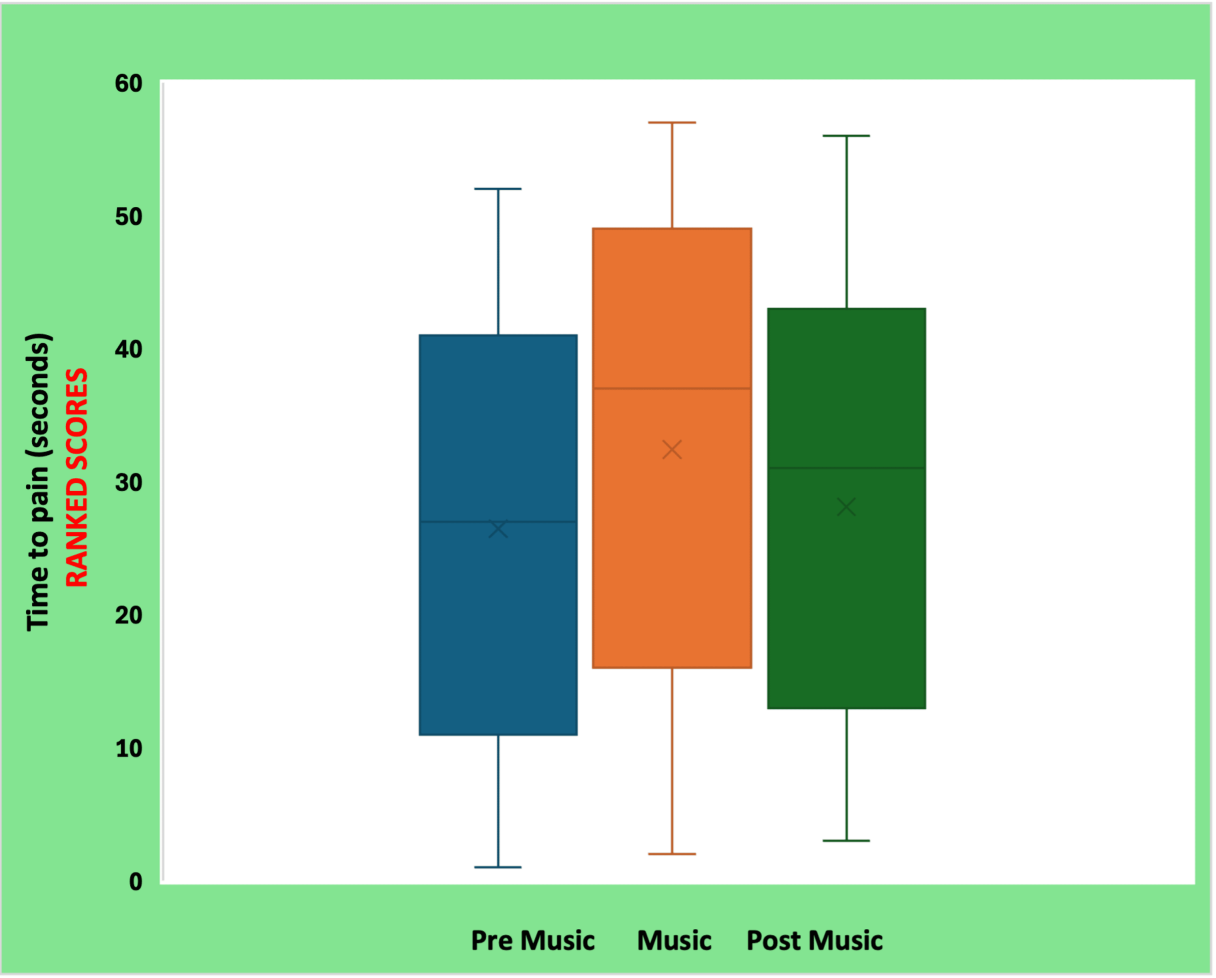
Ranked time-to-pain scores across pre-music, preferred music, and post-music conditions (Friedman test)

The ranked transformations used for non-parametric comparison are presented in Figure 2, confirming the consistent elevation of tolerance during the preferred music condition. Overall, 17 of 20 participants (85%) increased their time-to-pain perception while listening to preferred music, indicating a robust within-person analgesic effect.

Pain intensity ratings

**Figure 3:**
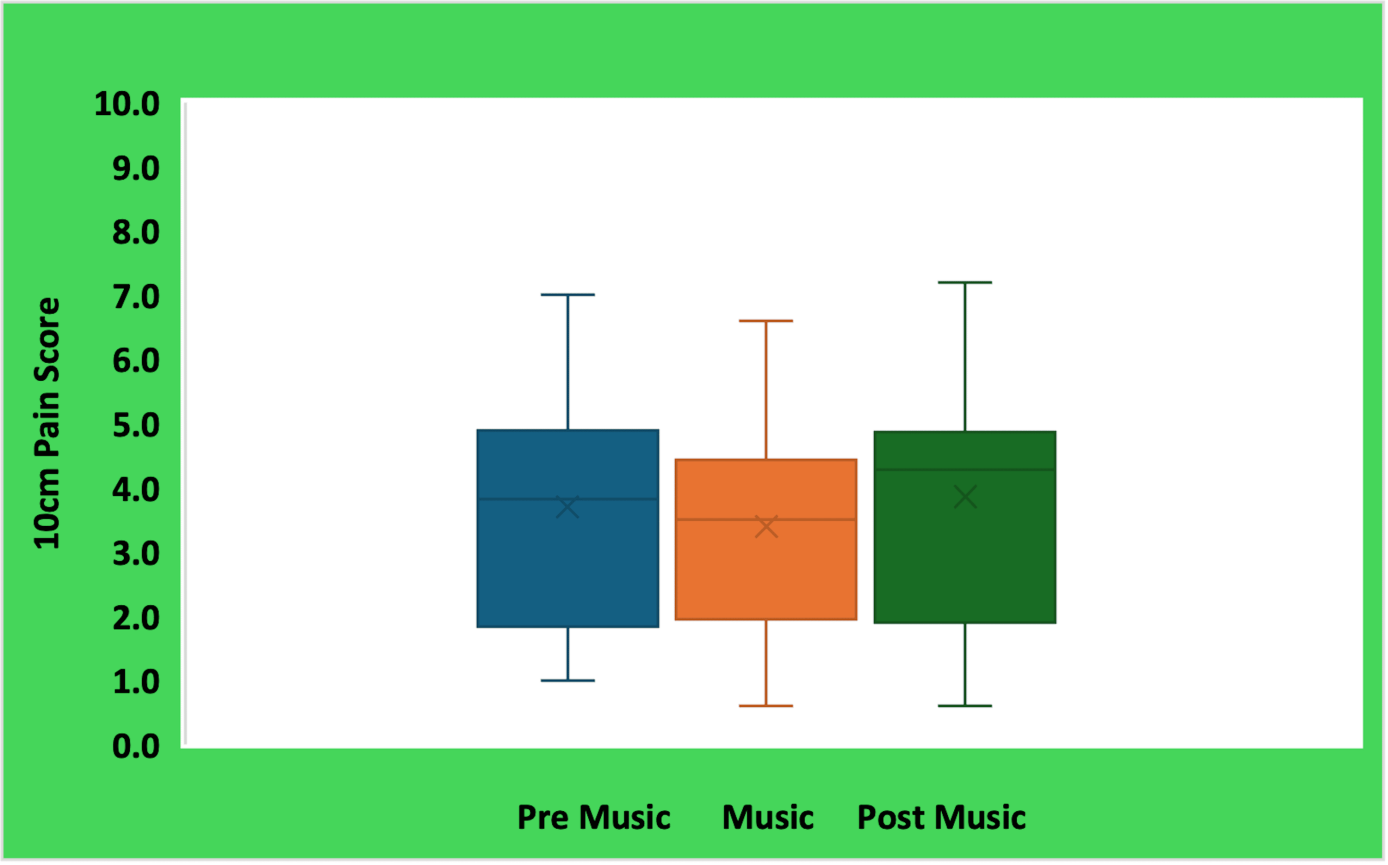
Pain intensity scores during pre-music, preferred music, and post-music conditions

Pain intensity scores were consistent across all three conditions. Mean VAS scores were 3.7 cm before music exposure, 3.4 cm during preferred music exposure, and 3.9 cm after music exposure. Because pain intensity ratings were normally distributed, a one-way repeated-measures ANOVA was conducted, revealing no significant effect of condition on subjective pain intensity (F(2, 38) = 0.561, p > 0.05). Pairwise comparisons using paired t-tests further confirmed that preferred music did not significantly alter perceived pain severity relative to either baseline or post-music conditions. Thus, although preferred music increased time-to-pain onset, it did not meaningfully change the intensity of pain perceived at the moment of withdrawal (Figure 3).

## Discussion

Our pilot study investigated whether listening to preferred music could modulate pain perception during an experimentally induced cold pressor stimulus. Consistent with the emerging literature on music-induced analgesia [7-10,12,18,19], our findings demonstrate that preferred music significantly increases the time-to-pain perception compared to both pre-music and post-music conditions. However, preferred music did not significantly affect participants’ perceived pain intensity at the moment of hand withdrawal. Together, our findings suggest that while preferred music enhances pain tolerance, its effects may operate more through delaying the onset of pain rather than attenuating perceived peak pain.

The observed prolongation of pain tolerance aligns closely with findings from Garcia and Hand, Timmerman et al., and Van der Valk Bouman et al., all of whom demonstrated that self-selected music enhances pain thresholds in experimental contexts [4,9,10]. Notably, our results mirror Van der Valk Bouman et al., who reported increased pain tolerance in a large sample of healthy festival attendees exposed to the CPT while listening to preferred music [10]. The consistency across studies, despite variations in setting and participant demographics, suggests that preference-driven music selection may be a robust facilitator of pain modulation.

In contrast to some previous studies [4,5,7,9,10,12,20], our findings did not detect significant reductions in perceived pain intensity. Several factors may explain this discrepancy. First, our study asked participants to withdraw their hand at the first pain perception, not pain tolerance; thus, pain intensity scores likely captured an early, less variable portion of the nociceptive experience. Second, our smaller sample size may have limited sensitivity to detect small but meaningful changes in pain ratings. Third, methodological differences (such as the specific timing of pain ratings, ambient conditions, or characteristics of the preferred music) may have contributed to variability. Nevertheless, the lack of significant change in pain intensity is not unexpected, given that pain tolerance and pain intensity represent distinct dimensions within the multidimensional pain experience.

The neurophysiological mechanisms underlying music-induced analgesia remain incompletely understood but are increasingly attributed to interactions across affective, reward, attentional, and autonomic pathways [21]. Lu et al. (2021) describe compelling evidence for modulation of hypothalamic-pituitary-adrenal axis activity, reductions in cortisol and β-endorphin levels, and activation of reward circuitry including the nucleus accumbens, ventral tegmental area, and anterior cingulate cortex [11]. Preferred music, in particular, appears to produce stronger dopaminergic responses, potentially amplifying attentional distraction, emotional regulation, and stress reduction, mechanisms that can collectively influence pain perception. Our findings add to this literature by demonstrating that even in a small controlled setting, preferred music meaningfully alters behavioral pain responses.

From a rehabilitation perspective, our findings warrant some notable attention. Patients undergoing active rehabilitation frequently encounter pain during exercise, manual therapy, stretching, or repetitive functional movements. An intervention as simple as providing patient-selected music may help increase tolerance to discomfort, potentially improving adherence and allowing patients to engage more fully in therapeutic exercises. Although our study did not show changes in peak pain intensity, increased tolerance to early pain onset may allow patients to complete more repetitions or sustain therapeutic positions longer, thereby enhancing treatment efficacy.

Practical implications

We believe our study findings have meaningful implications for rehabilitation practice. Incorporating preferred music into therapy sessions may offer a simple yet effective strategy to enhance patients’ tolerance for therapeutic exercises, especially those that involve mild discomfort or require sustained physical effort. In clinical settings, creating opportunities for patients to use their own music (whether through personal headphones or by allowing music to be played during treatment) may help them better manage discomfort and remain engaged in therapy tasks that are typically challenging. These benefits may extend beyond the clinic. 

Pairing preferred music with home exercise programs could improve patients’ willingness to persist with prescribed activities, potentially enhancing adherence and overall therapeutic outcomes. Because the analgesic effects appear to be strongest when the music is self-selected, clinicians should encourage patients to choose music that evokes motivation, relaxation, or positive emotion. Empowering patients to integrate personally meaningful music into both clinical and at-home rehabilitation activities may therefore support more comfortable, sustained, and effective participation in their recovery process.

Study strengths and limitations**

Our study has several strengths that enhance confidence in its findings. The within-subject repeated-measures design minimized inter-individual variability, allowing each participant to serve as their own control and strengthening the ability to detect the influence of preferred music on pain responses. Secondly, our cold pressor protocol was tightly controlled, ensuring consistent temperature exposure and standardized timing across all trials, which reduced methodological variances and improved study reliability. Importantly, our study used self-selected preferred music rather than standardized playlists; this choice increased real-world relevance (ecological validity) and aligns with evidence suggesting that the analgesic effects of music are strongest when the music is personally meaningful.

However, we acknowledge several limitations in our study. First, the small sample size (n = 20) limits generalizability and may have reduced statistical power, particularly for detecting subtle differences in pain intensity. Additionally, because participants were instructed to withdraw at the moment of first pain perception, variability in pain intensity ratings may have been constrained, potentially obscuring condition-related differences. Our sample was relatively homogenous (i.e., primarily young to middle-aged individuals who may or may not be affiliated with the university, as the adverts were only placed within the institution), which may not reflect broader clinical populations. Third, one participant exhibited unusually high tolerance values, highlighting natural heterogeneity that a larger study would be better equipped to accommodate. Finally, the short-term, highly controlled laboratory setting does not fully capture the complexity of pain experiences encountered during real-world rehabilitation, where emotional, contextual, and functional factors interact. As such, our future inquiry would examine the role of preferred music during clinically relevant therapeutic activities, explore whether longer or repeated exposure enhances analgesic effects, and investigate whether music pairing can meaningfully improve adherence and comfort in home exercise programs, which could help clarify how music can be optimally integrated into rehabilitation practice to support patient engagement and reduce discomfort.

## Conclusions

Our pilot study provides preliminary evidence that preferred music can enhance pain tolerance during experimentally induced cold pressor pain. Participants demonstrated a significantly longer time-to-pain perception when listening to self-selected music, suggesting that preferred music may modulate early nociceptive processing even within brief, controlled exposures. Although no changes were observed in perceived pain intensity, the delayed onset of pain remains clinically meaningful, particularly in rehabilitation settings where discomfort often limits engagement and persistence with therapeutic activities. Our findings support preferred music as a simple, low-cost, and patient-centered adjunct that may help individuals better tolerate rehabilitative exercises or other mildly painful procedures. Future research with larger, more diverse samples and clinically relevant tasks is needed to clarify underlying mechanisms, assess the durability of effects, and evaluate its utility in routine rehabilitation practice. Integrating preferred music into therapeutic environments has the potential to enhance comfort, engagement, and overall patient experience.
